# Emodin Alleviates Sodium Taurocholate-Induced Pancreatic Acinar Cell Injury *via* MicroRNA-30a-5p-Mediated Inhibition of High-Temperature Requirement A/Transforming Growth Factor Beta 1 Inflammatory Signaling

**DOI:** 10.3389/fimmu.2017.01488

**Published:** 2017-11-06

**Authors:** Hong Xiang, Xufeng Tao, Shilin Xia, Jialin Qu, Huiyi Song, Jianjun Liu, Dong Shang

**Affiliations:** ^1^College (Institute) of Integrative Medicine, Dalian Medical University, Dalian, China; ^2^Department of General Surgery, The First Affiliated Hospital of Dalian Medical University, Dalian, China; ^3^College of Pharmacy, Dalian Medical University, Dalian, China; ^4^Clinical Laboratory of Integrative Medicine, The First Affiliated Hospital of Dalian Medical University, Dalian, China

**Keywords:** emodin, pancreatitis, pancreatic acinar cells injury, miR-30a-5p, HTRA1, inflammation

## Abstract

Pancreatitis is an inflammatory disease that is responsible for substantial morbidity and mortality, and it can induce pancreatic necrosis that starts within pancreatic acinar cells in severe cases. Emodin, a pleiotropic natural product isolated from the Chinese herb *Rheum palmatum L*., has effective anti-inflammatory activities. In this paper, we investigated the protective effects and molecular mechanism of emodin against sodium taurocholate (STC)-induced pancreatic acinar cells injury *in vitro* and *in vivo*; and the results showed that emodin could significantly alleviate STC-induced pancreatic acinar cells injury through decreasing trypsin, amylase and the release of inflammatory factors (tumor necrosis factor alpha, interleukin-1β, and interleukin-6). Also, we found that emodin could significantly downregulate the HTRA1, interleukin-33, myeloid differentiation primary response gene 88, TNF receptor-associated factor-6, and nuclear factor kappa-B protein levels, but upregulate the transforming growth factor beta 1 (TGF-β1) protein level. These results indicated that emodin alleviated pancreatic acinar cells injury mainly through inhibiting HTRA1/TGF-β1 signaling pathway, and this finding was further proved by the HTRA1 overexpression experiments. In addition, the inflammatory regulator microRNA-30a-5p (miR-30a-5p) was confirmed to be a transcriptional brake that controls the *HTRA1* gene through using a dual luciferase reporter assay, and it was upregulated by emodin in pancreatic acinar cells. Furthermore, the pancreatic protective effects and anti-inflammatory activities of emodin were all abrogated with both miR-30a-5p inhibitor *in vitro* and miR-30a-5p antagomir *in vivo*. Collectively, these results demonstrate that miR-30a-5p/HTRA1 are the target of emodin-mediated attenuation of pancreatic acinar cell injury in pancreatitis, thus providing the foundation for further development of this natural product for medical therapy.

## Introduction

Pancreatitis is a sterile inflammation of the pancreas predominantly triggered by gallstones and alcohol misuse that has a global incidence ranging from 13 to 45 cases per 100,000 individuals per year (1). Convincing evidence has shown that the inappropriate activation of trypsin and a subsequent acute inflammatory response are the integrated pathophysiological features of pancreatitis (2, 3). Trypsin activation in the initial period of the disease is a short-term process; however, the exaggerated inflammatory cascade, which is not only limited to the pancreas but also includes other tissues, persists for a longer period of time. It evolves from a mild acute pancreatitis progressing into the systemic inflammatory response syndrome and multiple organ dysfunction (4, 5). Recent mechanistic studies also indicate that the initial pancreatic injury starts within pancreatic acinar cells and plays a vital role in mediating disease severity (6, 7). Consequently, blocking the inflammatory cascade and pancreatic acinar cells injury may be a more rational strategy for the pancreatitis therapy.

Serine protease high-temperature requirement A (HTRA) family proteins have a highly conserved protease domain and one or more C-terminal PDZ domains that mediate protein interactions that are involved in cell stress events through the degradation of misfolded proteins in the cytoplasm (8). Previous studies have primarily focused on the involvement of HTRA1 in cerebral autosomal recessive arteriopathy with subcortical infarcts and leukoencephalopathy (9), age-related macular degeneration (10), malignancy (11), pregnancy-induced hypertension (12), and other diseases; however, the relationship between HTRA1 and inflammatory diseases is unknown. Recent studies have reported that HTRA1 can prevent pro-transforming growth factor beta 1 (TGF-β1) from maturing into TGF-β1, which serves as a regulatory checkpoint for limiting inflammation (13, 14). Therefore, the activation of HTRA1/TGF-β1-mediated inflammatory signaling might play an important role or partially involved in the occurrence and progression of pancreatic acinar cells injury.

MicroRNAs (miRNAs), small non-coding RNAs of 20–22 nucleotides, are important regulators that modulate the expression of more than half of the protein-coding genes through either cleavage or translational repression (15). Currently, some miRNAs have been directly or indirectly associated with the pancreatitis progression, and the aberrant expression of these molecules may lead to the alteration of pivotal physiological functions involved in inflammation infiltration (16–18). For example, activated AR42J rat pancreatic acinar cells enhanced nuclear factor kappa-B (NF-κB) activation in macrophages through the secretion of exosomes carrying differentially expressed miRNAs (19). Bone marrow-derived mesenchymal stem cells deliver miR-9, which targets the *NF-*κ*B1/p50* gene, to the damaged pancreas or peripheral blood mononuclear cells, possibly attenuating severe acute pancreatitis (20). Thus, manipulating the functions of miRNAs is a potential therapeutic strategy to interfere with pancreatic acinar cells injury pathogenesis at the genetic level.

Emodin (1,3,8-trihydroxy-6-methylanthraquinone) is a natural anthraquinone derivative isolated from the Chinese herb *Rheum palmatum L*., and it has been utilized both alone and as a principal component in Chinese herbal formulas (e.g., Dachengqi and Qingyi decoctions) to treat acute pancreatitis in China for many years (5, 21–23). However, the precise mechanisms and drug targets underlying the protective effects have not been completely elucidated. Previous studies also indicate that emodin inhibits the proliferation and metastasis of various types of malignant tumors *via* alterations in the expression of miRNAs and their downstream target genes in cancer cells (24, 25); however, to the best of our knowledge, no studies have reported similar effects in inflammatory diseases.

In the present study, we speculated that the mechanism by which emodin inhibits the HTRA1/TGF-β1 signaling pathway involves a direct suppression of one or more miRNAs that target HTRA1 mRNA transcripts. Using bioinformatic analyses, we identified miRNA-30a-5p (miR-30a-5p) to be an important transcriptional brake that curbs *HTRA1* gene expression. Moreover, we have shown that miR-30a-5p is downregulated in pancreatic acinar cells stimulated with sodium taurocholate (STC) prior to the activation of the HTRA1/TGF-β1 signaling pathway, but emodin could reverse this process. Consistently, loss of function studies using inhibitors further defined miR-30a-5p as a potent candidate target of emodin, attenuating pancreatic acinar cells injury *via* the suppression of the HTRA1/TGF-β1 inflammatory signaling pathway. Collectively, these results demonstrate that the development of novel, natural targeted miRNA therapies is promising for future medical interventions.

## Materials and Methods

### Reagents and Materials

Ham’s F-12K medium and fetal bovine serum were purchased from the American Type Culture Collection (ATCC, Manassas, VA, USA). STC was obtained from Sigma Chemical Co. (St. Louis, MO, USA). Emodin was purchased from Solarbio Science and Technology Co. (Beijing, China). Rhodamine 110, bis-(CBZ-L-isoleucyl-l-prolyl-l-arginine amide) dihydrochloride (BZiPAR), and the EnzChek^®^ Ultra Amylase Assay Kit were purchased from Molecular Probes^®^ Labeling and Detection Technologies (Waltham, MA, USA). Tumor necrosis factor alpha (TNF-α), interleukin-1 beta (IL-1β), and interleukin-6 (IL-6) enzyme-linked immunosorbent assay (ELISA) kits were obtained from Lengton Biotech (Shanghai, China). RNAiso Plus reagent, PrimeScript^®^ RT reagent and SYBR^®^ PremixEx Taq™ II (TliRNaseH Plus) were purchased from TaKaRa Biotechnology Co., Ltd. (Dalian, China). The lactate dehydrogenase (LDH) assay kit was obtained from the Nanjing Jiancheng Institute of Biotechnology (Nanjing, China). The protein extraction kit was purchased from KEYGEN Biotech. Co., Ltd. (Nanjing, China). The bicinchoninic acid (BCA) protein assay kit was purchased from the Beyotime Institute of Biotechnology (Shanghai, China). Hoechst 33342 was obtained from Solarbio (Beijing, China). The Double-Luciferase Reporter Assay Kit was obtained from TransGen Biotech (Beijing, China). HTRA1-WT/MUT plasmid, lipofectamine 2000, and miR-30a-5p mimic and inhibitor were obtained from RiboBio Co., Ltd. (Guangzhou, China). The HTRA1-pcDNA3.1 plasmid, TGF-β1-pGL3-Basic, and miR-30a-5p antagomir were purchased from GenePharma Corp. (Shanghai, China). Rabbit anti-HTRA1, TGF-β1, interleukin-33 (IL-33), myeloid differentiation primary response gene 88 (MyD88), TNF receptor-associated factor-6 (TRAF-6), and NF-κB, horseradish peroxidase-conjugated goat anti-rabbit IgG, and TRITC-conjugated goat anti-rabbit IgG (H + L) were purchased from the Proteintech Group (Chicago, IL, USA). Mouse anti-monoclonal myeloperoxidase (MPO) antibody (FITC) was obtained from Abcam (Cambridge, UK).

### Cell Culture

AR42J rat pancreatic acinar cells (ATCC^®^ CRL-1492™), purchased from ATCC, were maintained in Ham’s F-12K medium supplemented with 20% fetal bovine serum in a humidified atmosphere of 5% CO_2_ at 37°C.

### STC-Induced Cell Injury

Cells were cultured in 96-well plates at a density of 1 × 10^5^ cells⋅mL^−1^ for 48 h and were then challenged with various concentrations of STC (16,000, 8,000, 4,000, 2,000, 1,000, 500, 250, 125, and 62.5 µM) for 1 h. Cell viability was assessed to determine the most suitable concentration of STC using the MTT method (26). Briefly, the plates were incubated with MTT solution (5 mg⋅mL^−1^) for 4 h at 37°C, and subsequently, 100 µL of dimethyl sulfoxide was added to dissolve the formazan crystals. Finally, the absorbance was measured at 490 nm using a microplate reader (Thermo, Waltham, MA, USA); and the data were performed log transformation in order to generate half maximal inhibitory concentration (IC50) curve for parametric analysis.

### Emodin Toxicity Assay

AR42J cells were cultured in 96-well plates at a density of 1 × 10^5^ cells·mL^−1^ for 48 h and were then treated with various concentrations of emodin (80, 60, 40, and 20 µM) for 2 h at 37°C. Subsequently, the cytotoxicity of this compound was assayed through the detection of released LDH at 450 nm using a microplate reader (Thermo, Waltham, MA, USA) according to the manufacturer’s instructions.

### Cell Viability and Morphology Assays

AR42J cells were cultured in 96-well plates at a density of 1 × 10^5^ cells·mL^−1^ for 48 h and then pretreated with various concentrations of emodin (40, 20, 10, 5, 2.5, 1.25, and 0.625 µM) for 2 h before being challenged with STC (498.2 µM) for 1 h. The cells in the model group were cultured without emodin, and the cells in the control group were cultured under normal conditions. The MTT method was used to assay cell viability as described above. Furthermore, AR42J cells were grown in six-well plates and treated with various concentrations of emodin (10, 20, and 40 µM) for 2 h at 37°C before being challenged with STC (498.2 µM) for 1 h; cell morphology was then imaged using a phase contrast microscope (Nikon, Chiyoda pill, Tokyo, Japan).

### Intracellular Proteinase Assays

In preparation for intracellular proteinase assays with rhodamine 110-based substrates, AR42J cells were incubated with HEPES-buffered saline (HBS; 5 mM HEPES and 0.15 M NaCl, pH 7.35) containing 2 mM EDTA (HBS-EDTA) and stored at 4°C for no longer than 2 h after removing the different media. Subsequently, the cells were incubated for approximately 20 min in the presence of 10 µM substrates. Finally, trypsin activity was investigated using laser confocal microscopy with 400× magnification (Leica, Wetzlar, Germany).

An EnzChek amylase assay kit was used to determine the quantitative kinetic activity of α-amylase in the supernatant samples from all of the AR42J cell cultures. Fifty microliters of each sample were added into 200 µg·mL^−1^ DQ starch substrate solution in a 96-well plate according to the EnzCheck protocol. The samples were incubated at room temperature, protected from light, for an appropriate amount of time (10–30 min). The relative fluorescence intensity was read at 505 nm using a multifunctional microplate reader (BioTek, Winooski, VT, USA).

### Immunofluorescence Detection of HTRA1

AR42J cells were cultured in six-well plates for 48 h and then treated with different media. Immunofluorescence measurements of HTRA1 were performed as previously described (26). The cells were incubated with diluted HTRA1 antibody (1:100) overnight, followed by TRITC-conjugated goat anti-rat IgG (H + L) for 1 h at 37°C; subsequently, the cells were restained with Hoechst 33342 (1 µg·mL^−1^) for 5 min. Digital images were collected using an Olympus BX63 fluorescence microscope (Olympus; Tokyo, Japan) at 200× magnification. The fluorescence intensity of the images was quantified using image-Pro Plus 6.0 software (Media Cybernetics, Inc., Rockville, MD, USA).

### Overexpression of the *HTRA1* Gene

AR42J cells were seeded onto six-well plates (1 × 10^5^ cells·mL^−1^) in a serum-free medium and transfected with HTRA1-pcDNA3.1 mixed with lipofectamine 2000 according to the manufacturer’s instructions. In addition, 24 h after transfection, the cells were cultured in the presence or absence of emodin (40 µM) for 2 h before being challenged with STC (498.2 µM) for an additional 1 h. Subsequently, the protein levels of HTRA1, TGF-β1, IL-33, MyD88, TRAF-6, and NF-κB; the mRNA levels of TNF-α, IL-1β, and IL-6; and the supernatant contents of amylase, lipase, TNF-α, IL-1β, and IL-6 were measured.

### Identification of Prospective miRNAs Upstream of HTRA1 *via* Bioinformatic Approaches

MicroRNAs are important regulators of gene expression. To elucidate the potential mechanism by which emodin protects against pancreatitis, candidate upstream miRNAs that target rat HTRA1 mRNA were identified in the miRanda, miRDB, TargetScan, and CLIP databases. The miRNAs from the four databases were further screened, and the resulting candidate miRNAs listed in Table S1 in Supplementary Material were predicted by several software programs. Furthermore, the TargetScan 7.1 database predicted that bases 366–373 in the 3′-UTR of HTRA1 constitute a possible binding site for miR-30a-5p.

### Dual Luciferase Reporter Assay

For the dual luciferase reporter assay, the 3′-UTR of HTRA1 was cloned into the pmiR-RB-REPORT™ vector to construct the wild-type (WT) 3′-UTR. Site-directed mutagenesis of the miR-30a-5p binding site in the HTRA1 3′-UTR was performed to make a mutant (MUT) 3′-UTR. Plasmids containing the WT miR-30a-5p-HTRA1 response element (HTRA1-WT) and the corresponding mutant (HTRA1-MUT) were purchased from RiboBio Co., Ltd. (Guangzhou, China). AR42J cells were co-transfected with plasmid DNA (HTRA1-WT or HTRA1-MUT) and miR-30a-5p mimic (50 nM) or the negative control for 24 h. The sequences of the miR-30a-5p mimic are listed in Table S2 in Supplementary Material. Luciferase activities were then assessed using a Double-Luciferase Reporter Assay Kit with a multifunctional microplate reader (BioTek, Winooski, VT, USA), normalized to the ratio of Firefly and Renilla luciferase signals. Furthermore, 24 h after transfection with HTRA1-WT/MUT plasmids and miR-30a-5p mimic, respectively, the HTRA1 protein level in AR42J cells was detected by western blotting to confirm whether HTRA1 is the target protein of miR-30a-5p.

In addition, plasmids containing HTRA1 (HTRA1-pCDNA3.1) and the TGF-β1 promoter (TGF-β1-pGL3-Basic) were purchased from GenePharma Inc. (Shanghai, China). HTRA1-pCDNA3.1 or TGF-β1-pGL3-Basic and miR-30a-5p mimic/inhibitor were co-transfected into AR42J cells for 24 h. The sequences of the miR-30a-5p inhibitor are listed in Table S2 in Supplementary Material. The dual luciferase reporter assay was then conducted as described above.

### Quantification of miR-30a-5p

Total miRNA from AR42J cells treated with or without emodin was extracted using an EasyPure^®^ miRNA Kit (TransGen Biotech, China) according to the manufacturer’s instructions. Reverse transcription was performed using the TransScript^®^ One-Step gDNA Removal and cDNA Synthesis SuperMix (TransGen Biotec, Beijing, China) according to the manufacturer’s instructions in a TC-512 PCR system (TECHNE, Cambridge, UK), and the levels of mature miRNA were quantified using real-time PCR with the TransScript^®^ Top Green qPCR SuperMix (TransGen Biotec, Beijing, China) in an ABI 7500 Real-Time PCR System (Applied Biosystems, Foster, CA, USA), and the U6 small nucleolar RNA was used for normalization. The pre-primers and post-primers (RN: R10031.4) of miR-30a-5p and U6 were obtained from RiboBio Co., Ltd. (Guangzhou, China).

### miR-30a-5p Inhibitor Treatment

AR42J cells were seeded onto 6-well plates (1 × 10^5^ cells·mL^−1^) in a serum-free medium and transfected with miR-30a-5p inhibitor (100 nM) mixed with lipofectamine 2000 for 24 h according to the manufacturer’s instructions. miR-30a-5p inhibitor sequence is listed in Table S2 in Supplementary Material. After transfection, the cells were treated with different media, and subsequently, the protein levels of HTRA1, TGF-β1, IL-33, MyD88, TRAF-6, and NF-κB, the mRNA levels of TNF-α, IL-1β, and IL-6, and the supernatant contents of amylase, lipase, TNF-α, IL-1β, and IL-6 were measured.

### Animals

All animal care and experimental procedures in the present study were approved by the Animal Care and Use Committee at Dalian Medical University and were performed in strict compliance with the People’s Republic of China Legislation Regarding the Use and Care of Laboratory Animals. Rats were obtained from the Experimental Animal Center at Dalian Medical University (Dalian, China) and maintained with a 12-h light–dark cycle in a temperature-controlled (25 ± 2°C) room. All the animal studies complied with the principle for replacement, refinement, or reduction (the 3Rs). The animals were housed in groups with three rats per cage with free access to standard laboratory food and water, and they were acclimatized for 1 week prior to the experiment.

### Study Design

Thirty male Sprague-Dawley (SD) rats weighing 180–220 g (6–8 weeks old) were randomly divided into five groups (*n* = 6, where “*n*” refers to the number of animals in each group): Group I, sham operation group (control); Group II, pancreatitis model group; Group III, emodin-treated group (60 mg·kg^−1^); Group IV, antagomir group; and Group V, antagomir + emodin group. Using an antagomir, a synthetically derived oligonucleotide sequence that complementary binds to the miRNA, it was able to inhibit miR-30a-5p activity and alleviate rejection in rats. And, the miR-30a-5p antagomir sequence is listed in Table S2 in Supplementary Material. Rats in the antagomir and antagomir + emodin groups were injected with antagomir (20 µmol·kg^−1^·day^−1^) in the tail vein for 3 days, and experimental models were established 1 h after injection on the last day as previously described (27). Briefly, animals were fasted for 12 h with free access to water and then anesthetized. Pancreatitis was induced by standard retrograde infusion of freshly prepared 5.0% STC (0.1 mL per 100 g body weight) into the biliopancreatic duct. The control group received a retrograde infusion of an equivalent volume of sterile saline. After establishing the animal model, emodin was intragastrically administered to the rats in the emodin and antagomir + emodin groups immediately and again after 6 h. Both the control and model groups were administered an equivalent volume of 0.5% carboxymethylcellulose sodium (CMC-Na). Blood samples were collected *via* the abdominal aorta of the rats for biochemical analyses after 12 h of duct infusion and anesthesia. Pancreatic head samples were fixed in 10% buffered formalin and embedded in paraffin for histopathological examination and immunofluorescence examinations. The other pancreas samples were immediately frozen and maintained at −80°C for real-time PCR and western blotting analyses.

### Histological and Immunofluorescence Assays

Formalin-fixed tissue samples from all of the groups were stained with hematoxylin and eosin (HE) and examined using light microscopy (Leica, Wetzlar, Germany) at 200× magnification. Typical images were collected, and the effects of emodin on pancreatic edema, inflammation, and vacuolization were evaluated. We applied a scoring system as shown in Table S3 in Supplementary Material.

Immunofluorescence localization of MPO and HTRA1 was performed with pancreas sections using previously described methods (27). After the slides were treated with mouse anti-MPO antibody (FITC) and rabbit anti-HTRA1 antibody overnight, they were then incubated with TRITC-conjugated goat anti-rabbit IgG (H + L) for 1 h at 37°C. Subsequently, the cells were restained with Hoechst 33342 (1 µg·mL^−1^) for 5 min. Digital images were obtained using an Olympus BX63 fluorescence microscope (Olympus BX63, Japan) at 200× magnification.

### Measurement of Enzyme and Inflammatory Cytokine Levels

The supernatant and plasma were collected from AR42J cells and rats after the experiments. Amylase, lipase, TNF-α, IL-1β, and IL-6 levels in the supernatant or plasma were assayed with ELISA kits according to the manufacturer’s instructions.

### Quantitative Real-time PCR Assay for TNF-α, IL-1β, and IL-6

Total RNA from AR42J cells and rat pancreas samples was extracted using the RNAiso Plus reagent according to the manufacturer’s instructions. Quantitative real-time PCR assay was conducted as described above. The primer sequences are listed in Table S4 in Supplementary Material. All gene expression levels were normalized to GAPDH, and the fold changes between the different groups were calculated using a standard curve for quantitative analysis.

### Western Blotting Assay

Total protein was extracted from AR42J cells and pancreas tissues using cold lysis buffer (containing 20 mM Tris, pH = 7.5, 150 mM NaCl, 1% Triton X-100, sodium pyrophosphate, β-glycerophosphate, EDTA, Na_3_VO_4_ and leupeptin) supplemented with 1 mM phenylmethyl sulfonyl fluoride, and their protein concentrations were determined using a BCA protein assay kit. The samples were separated using SDS-PAGE (10–15%) and transferred onto PVDF membranes (Millipore, USA). The membranes were blocked and incubated overnight at 4°C with primary antibodies for HTRA1, TGF-β1, IL-33, MyD88, TRAF-6, and NF-κB. The membranes were then incubated at room temperature with secondary antibodies, and protein expression was visualized using a ChemiDoc XRS Bioimaging System (Bio-Rad, USA). To eliminate variations in protein expression, the data were adjusted to the expression level of the corresponding internal reference [the integrated optical density (IOD) value of target protein versus the IOD of GAPDH].

### Data and Statistical Analysis

The data were expressed as the mean ± SD from three or more independent experiments. Statistical analysis was performed with GraphPad Prism 6.0 software (Graphpad Software, Inc., San Diego, CA, USA). One-way ANOVA/Kruskal–Wallis tests followed by *post hoc* Tukey’s/Dunn’s (according to homogeneity of variances: yes/no) were used for the analysis of the differences between multiple independent groups, and the unpaired *t*-test/Mann–Whitney *U* test was carried out for two different groups’ comparison (according to homogeneity of variances: yes/no). *Post hoc* tests were performed when ANOVAs indicated that a significant difference existed between groups. *p* values of <0.05 or <0.01 were considered statistically significant.

## Results

### Emodin Alleviates STC-Induced AR42J Cell Injury and Inflammation

**Figure 1:**
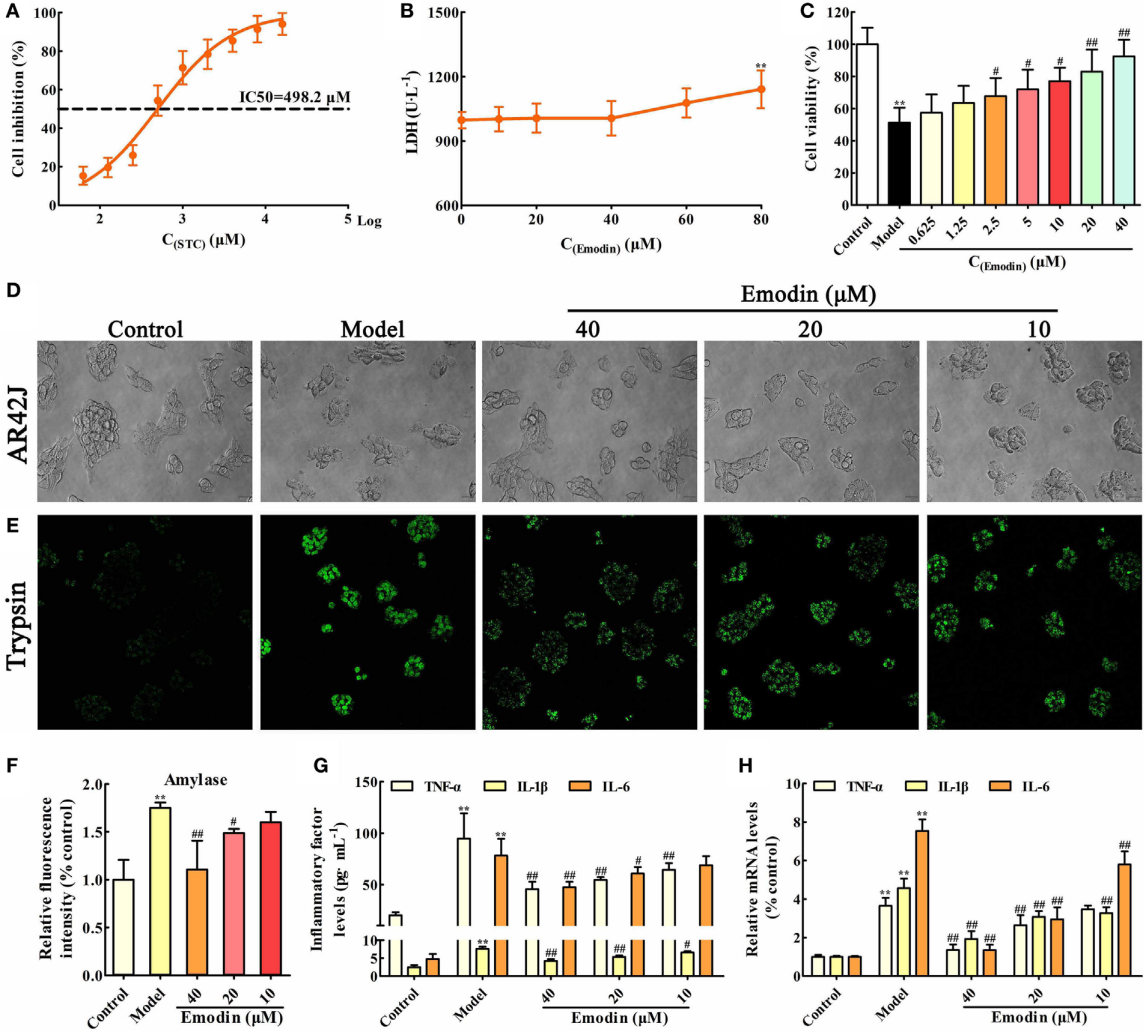
Emodin alleviates sodium taurocholate (STC)-induced AR42J cell injury and inflammatory reaction. **(A)** The half maximal inhibitory concentration (IC50) of STC on AR42J cells viabilities; **(B)** cytotoxicity of emodin on AR42J cells; **(C)** effects of different concentrations of emodin (0.625–40 µM) on the viabilities of STZ-induced AR42J cells; **(D)** effects of emodin (40, 20, and 10 µM) on the morphology and structure of STZ-induced AR42J cells (bright-field image, 400× magnification); **(E)** effects of emodin (40, 20, and 10 µM) on trypsin level based on the immunofluorescence assay (400× magnification); **(F)** effects of emodin (40, 20, and 10 µM) on amylase level based on the enzyme-linked immunosorbent assay (ELISA); **(G)** effects of emodin (40, 20, and 10 µM) on tumor necrosis factor alpha (TNF-α), interleukin-1β (IL-1β), and interleukin-6 (IL-6) levels in AR42J cells based on the ELISA; **(H)** effects of emodin (40, 20, and 10 µM) on TNF-α, IL-1β, and IL-6 mRNA levels in AR42J cells based on the RT-PCR assay. Data are presented as the mean ± SD (*n* = 6), where “*n*” refers to independent values. **p < 0.01 versus control group; ^#^*p* < 0.05 and ^##^*p* < 0.01 versus model group.

In order to choose the proper stimulus concentration, the viability of AR42J cells treated by different STC doses was detected through MTT assay, and the results showed that the IC50 of STC after 1 h treatment was 498.2 µM (Figure 1A), which was selected as the stimulus dose for the *in vitro* model. In addition, the cytotoxicity of emodin against AR42J cells was detected using LDH testing, and the results showed that 60 µM emodin had obvious cytotoxicity (*p* < 0.05); therefore, a maximum dose of 40 µM emodin was used in the following experiments (Figure 1B). As shown in Figure 1C, a dose of 498.2 µM STC significantly decreased the viability of the AR42J cells; however, doses of 2.5, 5, 10, 20, and 40 µM emodin notably increased cells viability in a dose-dependent manner. Moreover, the morphology of AR42J cells was observed using a light microscope, and the results revealed that STC significantly caused AR42J cell death and morphological changes characteristic for cell death, which was alleviated by emodin at doses of 40, 20, and 10 µM (Figure 1D). Trypsin is a pancreatitis marker that can be assayed *via* a rhodamine staining kit (19). Using a confocal laser-scanning microscope, we identified trypsin-positive cells with green color, and emodin at doses of 40, 20, and 10 µM significantly decreased the number of trypsin-positive cells (Figure 1E). In addition, amylase is another common biochemical marker of pancreatic disease (28). Through ELISA, it found that STC significantly induced the release of amylase, which was decreased by emodin in a dose-dependent manner (Figure 1F). Moreover, the protein and gene levels of inflammatory mediators were all detected by ELISA and real-time PCR, respectively. The results showed that TNF-α, IL-1β, and IL-6 were markedly increased in AR42J cells after treatment with STC; however, they were notably decreased by emodin at both the gene and protein levels (Figures 1G,H). Thus, these results proved that emodin could alleviate STC-induced AR42J cell injury and inflammation.

### Emodin Suppresses the HTRA1/TGF-β1 Signaling Pathway in STC-Injured AR42J Cells

**Figure 2:**
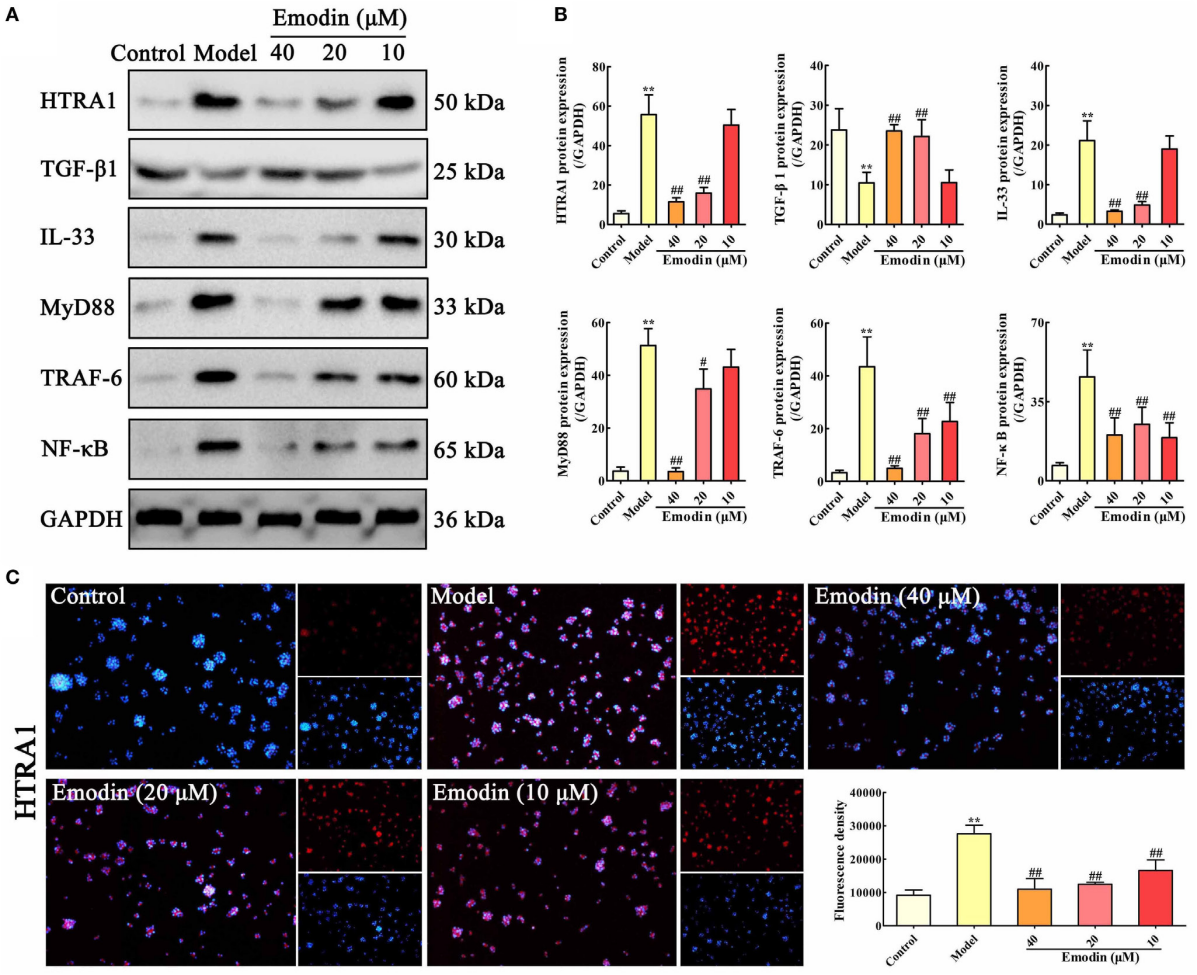
Emodin suppresses HTRA1/transforming growth factor beta 1 (TGF-β1) signaling pathway in sodium taurocholate (STC)-injured AR42J cells. **(A)** Effects of emodin (40, 20, and 10 µM) on HTRA1, TGF-β1, interleukin-33 (IL-33), myeloid differentiation primary response gene 88 (MyD88), TNF receptor-associated factor-6 (TRAF-6), and nuclear factor kappa-B (NF-κB) protein levels in STC-treated AR42J cells based on the western blotting assay. **(B)** Statistical analysis of the effects of emodin on protein expressions levels. Data are presented as the mean ± SD (*n* = 5), where “*n*” refers to independent values. **p < 0.01 versus control group;^#^ *p* < 0.05 and ^##^*p* < 0.01 versus model group. **(C)** Effects of emodin (40, 20, and 10 µM) on HTRA1 expression level in STC-treated AR42J cells based on the immunofluorescence assay (200× magnification).

In order to validate whether the HTRA1/TGF-β1 signaling pathway involves in the underlying mechanism of emodin against STC-induced AR42J cell injury, AR42J cells were treated with STC and different concentrations of emodin (40, 20, and 10 µM), and then the protein levels of HTRA1, TGF-β1, IL-33, MyD88, TRAF-6, and NF-κB were detected. The protein level of HTRA1 was significantly upregulated, and the protein level of TGF-β1 was markedly downregulated by STC compared with the control group; however, the alteration was reversed by emodin in a dose-dependent manner (Figures 2A,B). Subsequently, the levels of proteins downstream of the TGF-β1 inflammatory signal, including IL-33, MyD88, TRAF-6, and NF-κB, were all notably decreased by emodin compared to the STC-treated group. As previous method (29), the HTRA1 protein expression level was detected through the immunofluorescence assay, and the results indicated that STC notably increased the HTRA1 protein level (red fluorescence), which was markedly inhibited by emodin (40, 20, and 10 µM) in a dose-dependent manner (Figure 2C).

### Overexpression of HTRA1 Abrogates the Protective Effects of Emodin on STC-Injured AR42J Cells

**Figure 3:**
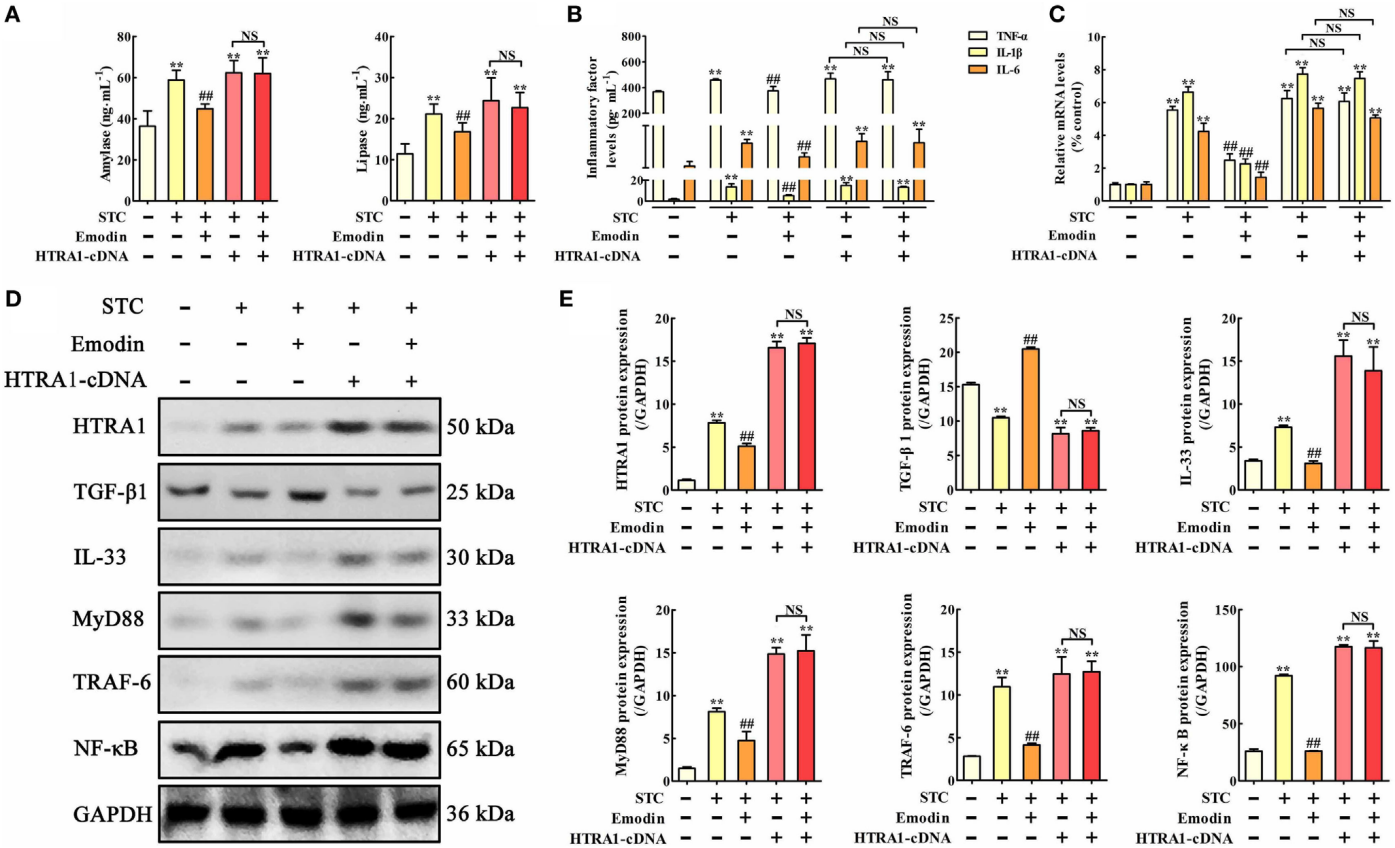
Protective effects of emodin on sodium taurocholate (STC)-injured AR42J cells are abrogated by HTRA1 overexpression *in vitro*. **(A)** Effects of emodin and HTRA1 overexpression on amylase and lipase levels in AR42J cells based on the enzyme-linked immunosorbent assay (ELISA); **(B)** effects of emodin and HTRA1 overexpression on tumor necrosis factor alpha (TNF-α), interleukin-1β (IL-1β), and interleukin-6 (IL-6) levels in AR42J cells based on the ELISA; **(C)** effects of emodin and HTRA1 overexpression on TNF-α, IL-1β, and IL-6 mRNA levels in AR42J cells based on the RT-PCR assay; **(D)** effects of emodin and HTRA1 overexpression on HTRA1, transforming growth factor beta 1 (TGF-β1), interleukin-33 (IL-33), myeloid differentiation primary response gene 88 (MyD88), TNF receptor-associated factor-6 (TRAF-6), and nuclear factor kappa-B (NF-κB) expression levels in STC-treated AR42J cells based on the western blotting assay; **(E)** statistical analysis of the effects of emodin on proteins expressions levels. Data are presented as the mean ± SD (*n* = 5), where “*n*” refers to independent values. **p < 0.01 versus control group; ^##^*p* < 0.01 versus model group; NS, not significant.

To explore the role of HTRA1 in the anti-inflammatory activity of emodin, an *in vitro* overexpression approach performed by HTRA1-pcDNA3.1 was used. As shown in Figure 3A, lipase and amylase levels in emodin-treated AR42J cells were significantly decreased compared with the STC group, but these inhibitory actions were significantly abrogated by HTRA1 overexpression regardless of the presence of emodin. Similar results were also observed at the protein and mRNA expression levels of TNF-α, IL-1β, and IL-6 (Figures 3B,C). In addition, as shown in Figures 3D,E, after transfecting with HTRA1-pcDNA3.1 regardless of the presence of emodin, the expression levels of HTRA1, IL-33, MyD88, TRAF-6, and NF-κB were all notably increased, and the TGF-β1 expression level was markedly decreased compared to the control group. These results suggested that overexpression of HTRA1 abrogated the regulation of the HTRA1/TGFβ1 signaling pathway by emodin.

### miR-30a-5p Targets HTRA1

**Figure 4:**
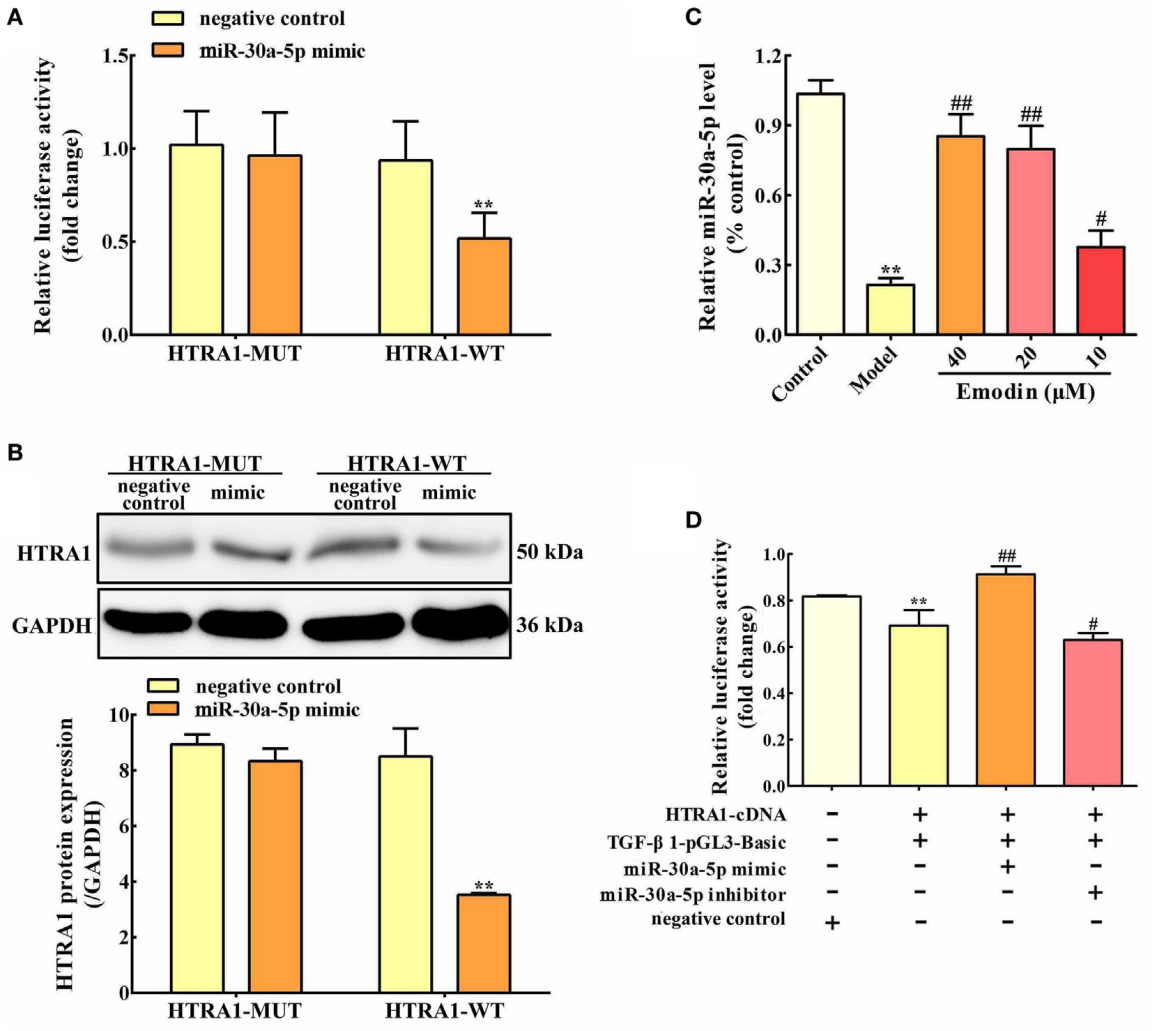
miR-30a-5p targets HTRA1 and the effects of emodin on the miR-30a-5p level in AR42J cells. **(A)** Dual luciferase reporter assay of HTRA1 and miR-30a-5p in AR42J cells; **(B)** effects of miR-30a-5p mimic on the HTRA1 protein expression levels based on western blot assay; **(C)** effects of emodin (40, 20, and 10 µM) on miR-30a-5p expressions in AR42J cells based on the RT-PCR assay; **(D)** effects of miR-30a-5p mimic and inhibitor on the inhibition of HTRA1 on transforming growth factor beta 1 (TGF-β1) based on the dual luciferase reporter assay. Data are presented as the mean ± SD (*n* = 5), where “*n*” refers to independent values. **p < 0.01 versus control group; ^#^*p* < 0.05 and ^##^*p* < 0.01 versus model group.

Based on bioinformatic approaches, we collected the prospective target miRNAs of HTRA1 and predicted that miR-30a-5p might be an important transcriptional brake that modulates targeted HTRA1 mRNA translation. The RNA sequence alignment showed that the 3′-UTR of HTRA1 mRNA contained a site complementary to the seed region of miR-30a-5p (Figure S1 in Supplementary Material). To further verify this finding, dual luciferase reporter assay was performed, and the results showed that luciferase activity in HTRA1-WT plasmid group was significantly repressed by miR-30a-5p mimic, but the effect was not observed in the HTRA1-MUT plasmid group (Figure 4A). In addition, western blot analysis further showed that miR-30a-5p mimic could markedly downregulate HTRA1 protein expression in HTRA1-WT plasmid group (Figure 4B). Therefore, these results showed that miR-30a-5p was likely to control target *HTRA1* gene expression, accordingly repressing protein translation. Furthermore, in order to determine whether emodin has a regulatory effect on miR-30a-5p, we detected the miR-30a-5p level through quantitative real-time PCR assay. As shown in Figure 4C, the results indicated that emodin at doses of 40, 20, and 10 µM significantly upregulated the expression levels of miR-30a-5p in AR42J cells compared with the model group. These results showed that the inhibition of emodin on HTRA1 maybe through activating miR-30a-5p expression.

### miR-30a-5p Affects the Inhibition of TGF-β1 by HTRA1

To validate the inhibition of TGF-β1 by HTRA1, we constructed TGF-β1 promoter dual luciferase plasmid (TGF-β1-pGL3-Basic) and performed the dual luciferase reporter assay. As shown in Figure 4D, the results showed that HTRA1 overexpression could markedly decrease the relative luciferase activity compared to the negative control, indicating that the HTRA1 protein significantly inhibits the TGF-β1 protein. Moreover, to observe the effects of miR-30a-5p on the inhibition of TGF-β1 by HTRA1, the AR42J cells were transfected with miR-30a-5p mimic and inhibitor, respectively. The results showed that miR-30a-5p mimic increased the luciferase activity, which was decreased by miR-30a-5p inhibitor (Figure 4D). The underlying mechanism involves in the miR-30a-5p-mediated inhibition of HTRA1 protein. Therefore, these results further proved that miR-30a-5p targeted *HTRA1* gene and affected the inhibition of TGF-β1 by HTRA1.

### miR-30a-5p Inhibitor Abrogates the Protective Effects of Emodin on STC-Injured AR42J Cells

**Figure 5:**
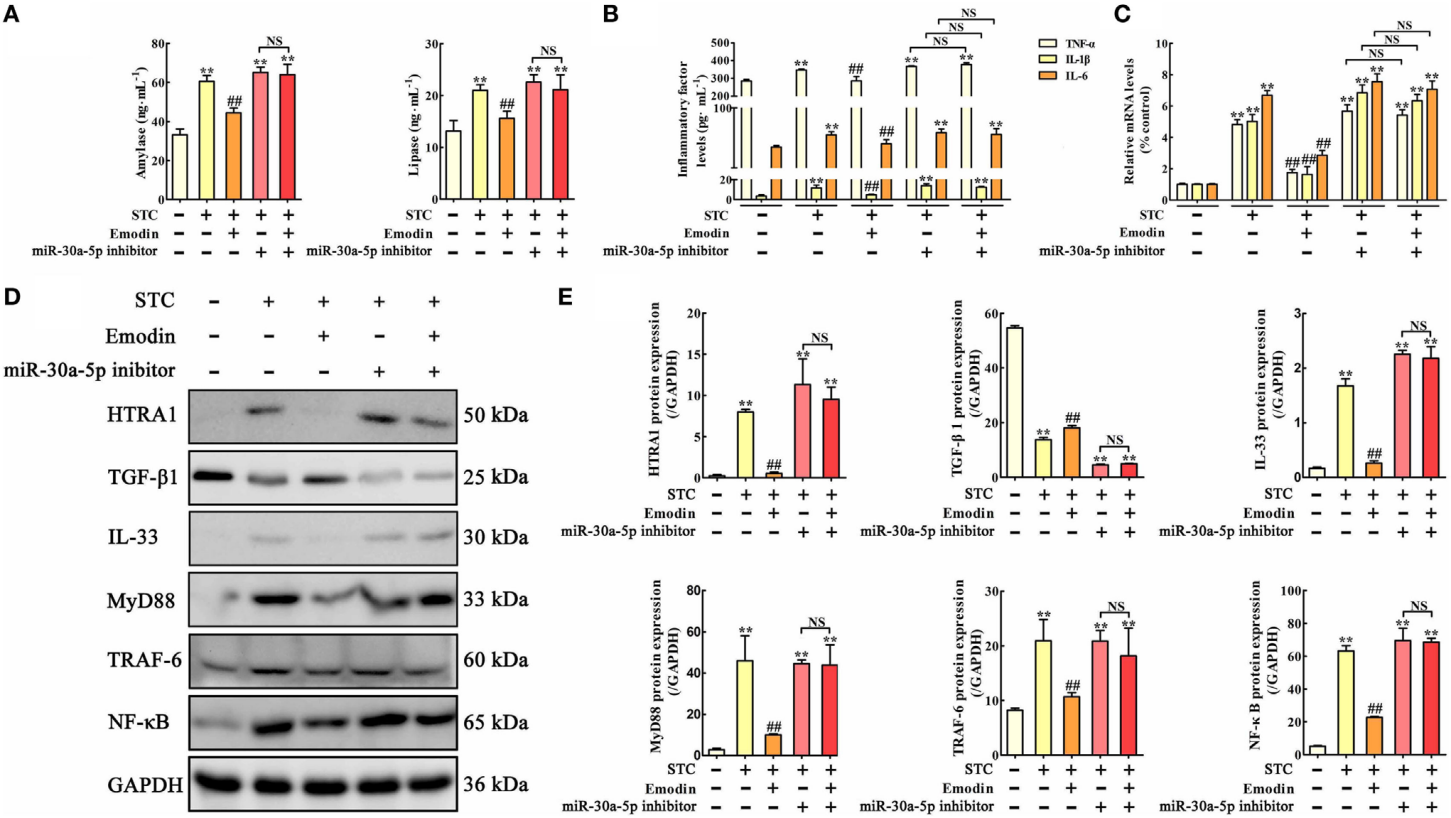
Protective effects of emodin on sodium taurocholate (STC)-injured AR42J cells were abrogated by miR-30a-5p inhibitor *in vitro*. **(A)** Effects of emodin and miR-30a-5p inhibitor on amylase and lipase levels in AR42J cells based on the enzyme-linked immunosorbent assay (ELISA); **(B)** effects of emodin and miR-30a-5p inhibitor on tumor necrosis factor alpha (TNF-α), interleukin-1β (IL-1β), and interleukin-6 (IL-6) levels in AR42J cells based on the ELISA; **(C)** effects of emodin and miR-30a-5p inhibitor on TNF-α, IL-1β, and IL-6 mRNA levels in AR42J cells based on the RT-PCR assay; **(D)** effects of emodin and miR-30a-5p inhibitor on HTRA1, transforming growth factor beta 1 (TGF-β1), interleukin-33 (IL-33), myeloid differentiation primary response gene 88 (MyD88), TNF receptor-associated factor-6 (TRAF-6), and nuclear factor kappa-B (NF-κB) expression levels in STC-treated AR42J cells based on the western blotting assay; **(E)** statistical analysis of the effects of emodin on protein expression levels. Data are presented as the mean ± SD (*n* = 5), where “*n*” refers to independent values. **p < 0.01 versus control group; ^##^*p* < 0.05 versus model group; NS, not significant.

*In vitro* miR-30a-5p inhibitor transfection approach was used to observe the regulatory role of miR-30a-5p in the anti-inflammatory activity of emodin. The results showed that amylase and lipase levels were significantly increased by miR-30a-5p inhibitor both in the presence or absence of emodin compared with the control group; therefore, the inhibitory effects of emodin on lipase and amylase were significantly abrogated by miR-30a-5p inhibitor (Figure 5A). The protein and mRNA expression levels of TNF-α, IL-1β, and IL-6 in AR42J cells showed similar results (Figures 5B,C). Furthermore, after transfection of the cells with the miR-30a-5p inhibitor or miR-30a-5p inhibitor plus emodin, the expression levels of the HTRA1, IL-33, MyD88, TRAF-6, and NF-κB proteins were all significantly upregulated and the TGF-β1 protein level was markedly downregulated compared with the control group (Figures 5D,E). These results suggest that the miR-30a-5p inhibitor abrogated the protective effects of emodin on STC-injured AR42J cells and the regulation of the HTRA1/TGFβ1 signaling pathway by emodin.

### Emodin Exerts Protective Effects against Pancreatitis in Rats

**Figure 6:**
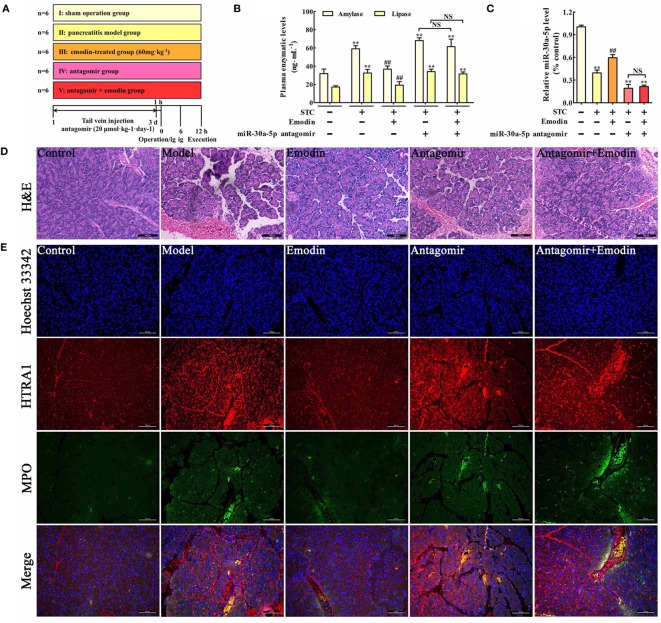
Emodin exerts the protective effects against pancreatitis in rats. **(A)** The study schema for the *in vivo* experiment on rats. **(B)** Effects of emodin (60 mg·kg^−1^) and miR-30a-5p antagomir on amylase and lipase levels in pancreatitis rats based on the ELISA; **(C)** effects of emodin (60 mg·kg^−1^) and miR-30a-5p antagomir on miR-30a-5p expression in pancreatitis rats based on the RT-PCR assay; **(D)** effects of emodin (60 mg·kg^−1^) and miR-30a-5p antagomir on pancreas histopathology based on H&E staining (200× magnification). **(E)** Effects of emodin (60 mg·kg^−1^) and miR-30a-5p antagomir on HTRA1/myeloperoxidase (MPO) double staining (200× magnification). Data are presented as the mean ± SD (*n* = 6), where “*n*” refers to independent values. **p < 0.01 versus control group; ^##^*p* < 0.05 versus model group; NS, not significant.

To further explore the role of miR-30a-5p in protective effects of emodin against rat pancreatitis, *in vivo* miR-30a-5p antagomir experiment was performed according to the study schema (Figure 6A). As shown in Figure 6B, the amylase and lipase levels in emodin (60 mg·kg^−1^)-treated rats were significantly decreased compared with the model rats, but these inhibitory actions were markedly abrogated in the miR-30a-5p antagomir group. Moreover, miR-30a-5p antagomir could markedly decrease the expression of miR-30a-5p regardless of the presence of emodin (Figure 6C). Furthermore, the histological architecture of pancreas tissue was investigated based on the HE staining (Figure 6D; Figure S2 in Supplementary Material). The results showed that pancreatic tissue injury of model group was characterized with acinar cell vacuolization, hemorrhage, leukocyte infiltration and necrosis; however, 60 mg·kg^−1^ of emodin could markedly decrease these pathological changes, which were abrogated by the miR-30a-5p antagomir. Therefore, miR-30a-5p plays an important role in the protective effects of emodin against rat pancreatitis.

### Emodin Decreases HTRA1 Expression and Suppresses Inflammation in Pancreatitis Rats

Myeloperoxidase is a classical biomarker of neutrophil activation and inflammation in pancreatitis (30). To examine the effects of emodin on HTRA1 expression and inflammation in pancreatic tissue, a double immunofluorescence assay was performed. As shown in Figure 6E, HTRA1 protein expression and the number of MPO-positive cells both significantly increased in the pancreatic tissue of rat pancreatitis compared with those in the control group, and this effect was decreased by emodin at a dose of 60 mg·kg^−1^. Moreover, both in the presence or absence of emodin after transfecting with the miR-30a-5p antagomir, HTRA1 protein expression and the number of MPO-positive cells were notably increased, suggesting that the miR-30a-5p antagomir reversed the actions of emodin on HTRA1 expression and inflammation. Therefore, emodin primarily inhibited inflammatory reactions *in vivo* by increasing miR-30a-5p.

### Emodin Suppresses the HTRA1/TGF-β1 Signaling Pathway by Increasing miR-30a-5p

**Figure 7:**
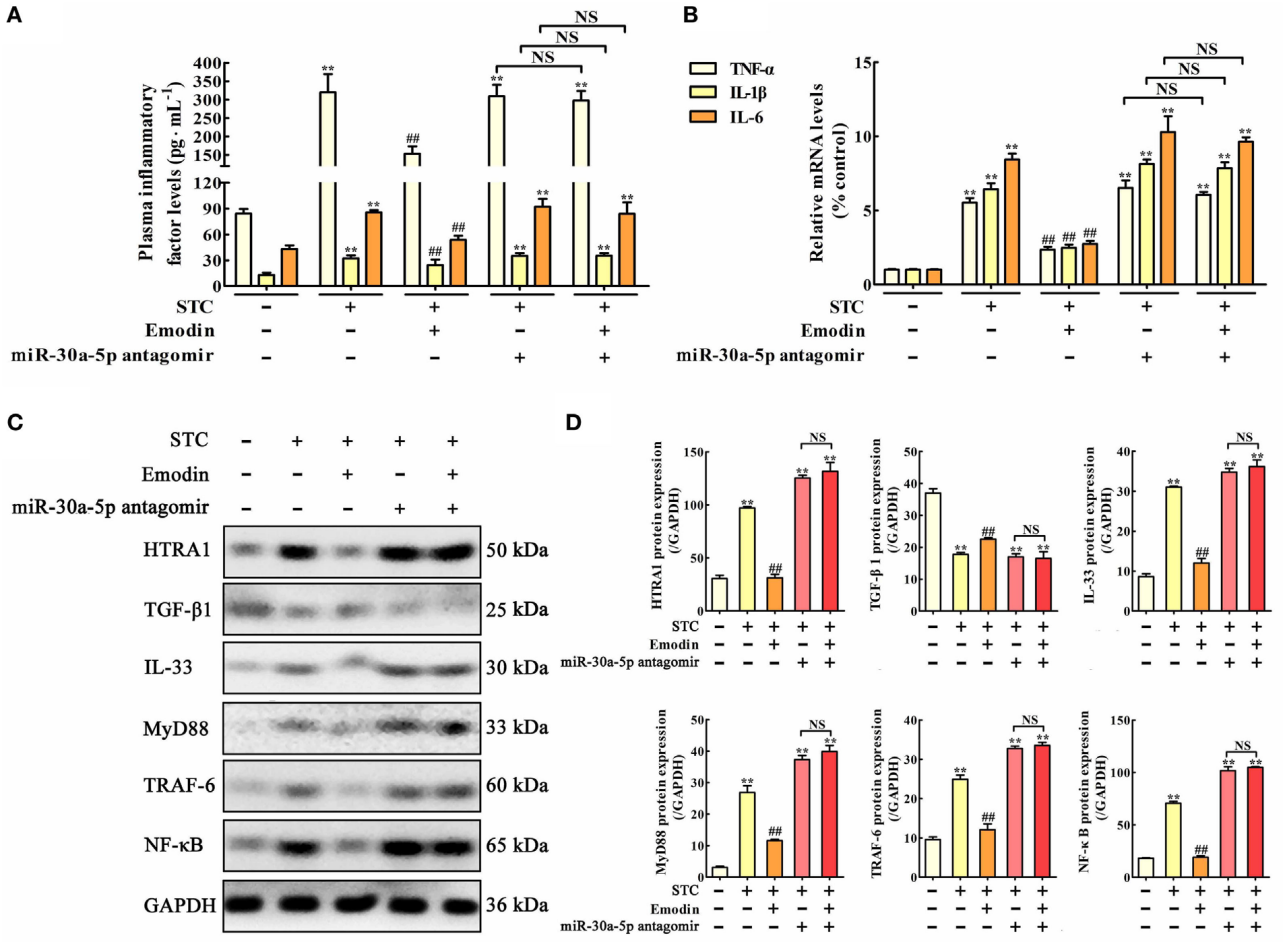
Protective effects of emodin on pancreatitis rats are abrogated by miR-30a-5p antagomir *in vivo*. **(A)** Effects of emodin and miR-30a-5p antagomir on tumor necrosis factor alpha (TNF-α), interleukin-1β (IL-1β), and interleukin-6 (IL-6) plasma levels of pancreatitis rats based on the enzyme-linked immunosorbent assay (ELISA); **(B)** effects of emodin and miR-30a-5p antagomir on TNF-α, IL-1β, and IL-6 mRNA levels in pancreas tissue of pancreatitis rats based on the RT-PCR assay; **(C)** effects of emodin and miR-30a-5p antagomir on HTRA1, transforming growth factor beta 1 (TGF-β1), interleukin-33 (IL-33), myeloid differentiation primary response gene 88 (MyD88), TNF receptor-associated factor-6 (TRAF-6), and nuclear factor kappa-B (NF-κB) expression levels in pancreas tissue of pancreatitis rats based on the western blotting assay; **(D)** statistical analysis of the effects of emodin on protein expressions levels. Data are presented as the mean ± SD (*n* = 5), where “*n*” refers to independent values. **p < 0.01 versus control group;^##^ *p* < 0.05 versus model group; NS, not significant.

In order to test the role of miR-30a-5p in the regulation of inflammation and HTRA1/TGF-β1 signaling pathway by emodin in rat pancreatitis, *in vivo* miR-30a-5p antagomir experiment was performed. Similar results were also observed both on the TNF-α, IL-1β, and IL-6 plasma levels (Figure 7A) and the expression levels of TNF-α, IL-1β, and IL-6 mRNA in pancreas tissue (Figure 7B). Moreover, after transfecting with the miR-30a-5p antagomir, inflammatory factors including TNF-α, IL-1β, and IL-6 were significantly increased regardless of the presence of emodin, suggesting that the miR-30a-5p antagomir reversed the anti-inflammatory activity of emodin in model rats. In addition, as shown in Figures 7C,D, the protein expression levels of HTRA1, IL-33, MyD88, TRAF-6, and NF-κB were all notably decreased, and the TGF-β1 expression level was markedly increased after treatment with emodin (60 mg·kg^−1^). However, these *in vivo* regulatory actions were all reversed by the miR-30a-5p antagomir. Therefore, miR-30a-5p silencing induced by its antagomir abrogated the anti-inflammatory activity of emodin in pancreatitis rats, which showed that miR-30a-5p exerted critical regulation in anti-inflammatory effect of emodin by regulating HTRA1/TGFβ1 signaling pathway.

## Discussion

Pancreatitis is a life-threatening disease that leads to acute hospitalization, and the reported global annual incidence of pancreatitis has increased (31). Previous extensive efforts in the development of a drug to treat pancreatitis were generally focused at the level of receptor/ligand, signaling, and transcription factor activation, which collectively coordinate the intensity and duration of pancreatitis (5, 32–35). In this study, we found that miR-30a-5p/HTRA1 may be a potent candidate target of the emodin-mediated attenuation of pancreatic acinar cells injury in pancreatitis, providing the foundation for the development of novel natural products for future medical interventions.

*In vitro*, we used the model of STC-injured AR42J rat pancreatic acinar cells and found that emodin at the no-cytotoxic concentration could significantly increase the viability and alleviate the morphological injury of AR42J cells after treatment with STC. *In vivo*, we used the rat model of STC-induced pancreatitis and further proved that emodin could markedly decrease pathological insult and neutrophil activation in pancreatic tissue. Moreover, emodin could notably decrease the release of pancreatitis markers (amylase and lipase) and inflammatory mediators, such as TNF-α, IL-1β, and IL-6. Therefore, these results proved that emodin significantly alleviate STC-induced pancreatic acinar cells injury through decreasing inflammatory reaction.

In addition, in order to identify the underlying molecular mechanism, we detected the effects of emodin on HTRA1/TGF-β1 signaling pathway. Previous studies have reported that HTRA1 can inhibit TGF-β1 protein level, which serves as a regulatory checkpoint for limiting inflammation through the suppression of IL-33 (13, 14). Furthermore, IL-33 pairing with ST2 receptors recruits and activates the MyD88 adapter protein concomitantly with TRAF-6, ultimately resulting in the activation of NF-κB and the release of inflammatory cytokines including TNF-α, IL-1β, and IL-6 (27, 36–38). Therefore, we determined the expression levels of these related proteins, and the results indicated that emodin could significantly downregulate the HTRA1, IL-33, MyD88, TRAF-6, and NF-κB protein levels, but upregulate the TGF-β1 protein level, and decrease the TNF-α, IL-1β, and IL-6 mRNA levels. In addition, HTRA1 overexpression abrogated the protective effect on pancreatic acinar cells and the regulation of the HTRA1/TGFβ1 signaling pathway by emodin. Together, these results showed that emodin had potent activity against STC-induced pancreatic acinar cells injury by controlling the HTRA1/TGF-β1 signaling pathway and its subsequent inflammatory reactions.

Furthermore, we found that miR-30a-5p is likely to control target *HTRA1* gene expression by binding to its 3′-UTR, accordingly repressing protein translation through using a dual luciferase reporter assay. In addition, previous studies have shown that HTRA1 may be a novel antagonist of TGF-β1 (13, 14). In this paper, we constructed TGF-β1 promoter dual luciferase plasmid to validate the inhibition of TGF-β1 by HTRA1, and the results showed that HTRA1-plasmid markedly decreased the relative luciferase activity. Moreover, miR-30a-5p mimic increased the TGF-β1 luciferase activity, which was decreased by the miR-30a-5p inhibitor, reflecting the miR-30a-5p-mediated inhibition on HTRA1. Experimental verification also revealed that miR-30a-5p is downregulated in AR42J cells and rat pancreatic tissue challenged with STC prior to the activation of the HTRA1 compared to the control group, and these alterations were reversed by emodin. Therefore, these results indicated that emodin could inhibit the HTRA1/TGF-β1 signaling pathway and its subsequent inflammatory responses by increasing miR-30a-5p.

Consistently, loss of function studies further defined miR-30a-5p as a potent candidate target of emodin that attenuates pancreatic acinar cells injury induced by STC *via* suppression of the HTRA1/TGF-β1 signaling pathway. Briefly, miR-30a-5p inhibitor and antagomir abrogated the anti-inflammatory activity of emodin *in vitro* and *in vivo*, respectively. These mechanistic studies proved that emodin exerted protective effects on pancreatitis by targeting miR-30a-5p.

**Figure 8:**
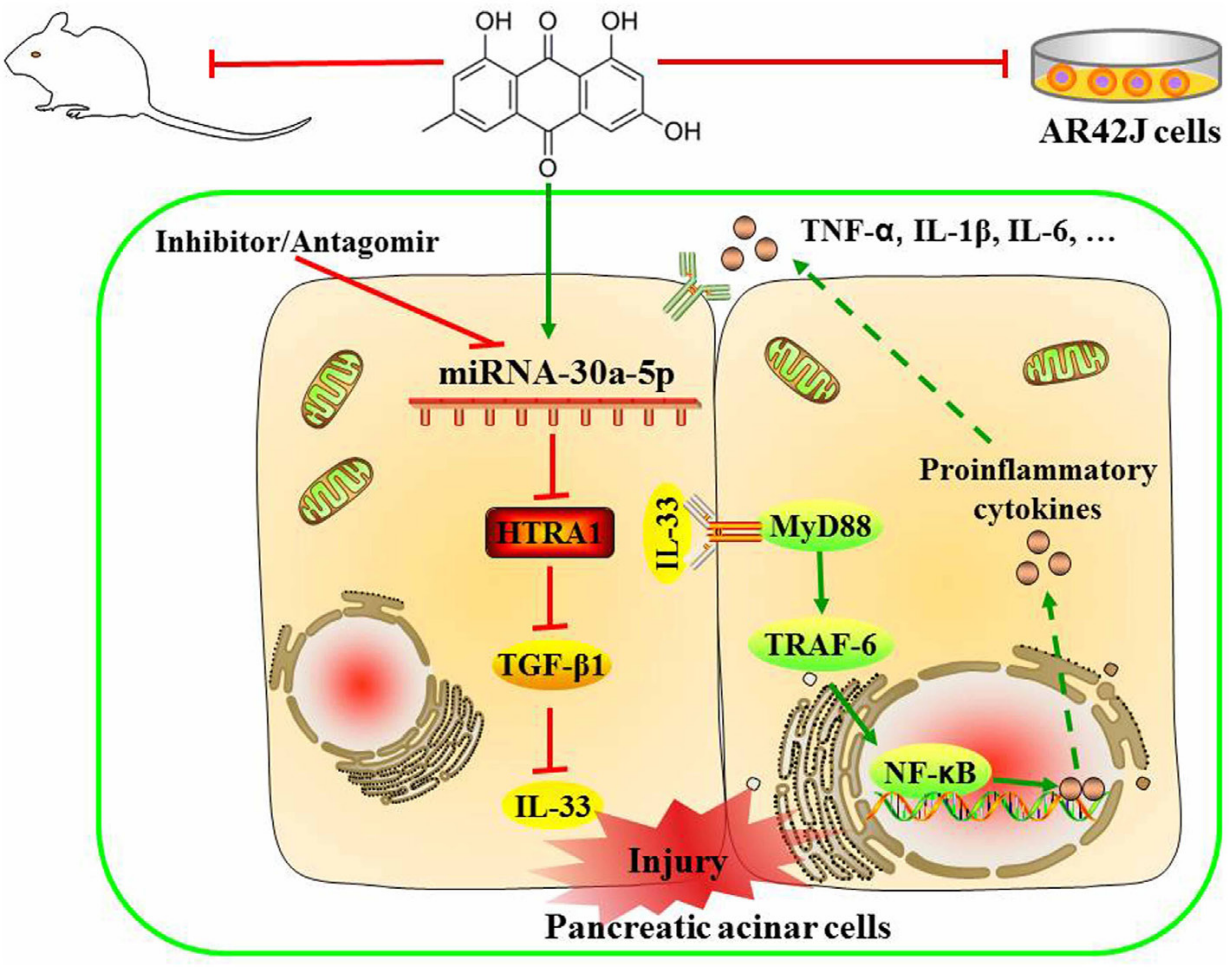
Proposed models for the effects of emodin against sodium taurocholate-induced pancreatic acinar cells injury.

In summary, as shown in Figure 8, we employed detailed molecular approaches to demonstrate that the emodin-mediated upregulation of miR-30a-5p expression in pancreatic acinar cells is essential for controlling inflammatory responses through the suppression of HTRA1/TGF-β1 signaling pathway activation. In briefly, emodin could significantly increase miR-30a-5p expression *in vitro* and *vivo*, and accordingly inhibit HTRA1 expression and lead to the activation of TGF-β1 anti-inflammatory pathway that mainly involved the downstream proteins including IL-33, MyD88, TRAF-6, and NF-κB. Finally, emodin significantly inhibits the release of proinflammatory factors (TNF-α, IL-1β, and IL-6). However, these actions of emodin are all abolished by both miR-30a-5p inhibitors *in vitro* and miR-30a-5p antagomir *in vivo*. Therefore, miR-30a-5p acts as a novel drug target of emodin to prevent spontaneous activation of the HTRA1/TGF-β1 signaling pathway, which has potential implications in pancreatitis pathologies. The insights obtained from this study further advance the development of emodin for future medical interventions.

## Author Contributions

HX and DS designed the experiments and drafted the manuscript. HX, XT, SX, and JQ performed the animal and cell culture experiments. HX, XT, HS, and JL performed the quantitative real-time PCR and western blotting assays. XT performed the gene transfection experiments. HX and DS edited the manuscript.

## Conflict of Interest Statement

The authors declare that the research was conducted in the absence of any commercial or financial relationships that could be construed as a potential conflict of interest.

## Abbreviations

BMSCs, bone marrow-derived mesenchymal stem cells; CARASIL, cerebral autosomal recessive arteriopathy with subcortical infarcts and leukoencephalopathy; CMC-Na, sodium carboxylmethyl cellulose; DMSO, dimethyl sulfoxide; HTRA1, serine protease high-temperature requirement A1; IL-1β, interleukin-1β; IL-6, interleukin-6; IL-33, interleukin-33; LDH, lactate dehydrogenase; MAP, mild acute pancreatitis; MPO, myeloperoxidase; MyD88, myeloid differentiation primary response gene 88; NF-κB, nuclear factor kappa-B; PBMCs, peripheral blood mononuclear cells; STC, sodium taurocholate; TGF-β1, transforming growth factor beta 1; TNF-α, tumor necrosis factor alpha; TRAF-6, TNF receptor-associated factor-6.

## References

[1] Lankisch PG Apte M Banks PA. Acute pancreatitis. Lancet (2015) 386:85–96. 10.1016/S0140-6736(14)60649-8 25616312

[2] Famularo G Minisola G De Simone C Acute pancreatitis. N Engl J Med (2006) 355:961; author reply 961. 10.1056/NEJMc061618 16943414

[3] Awla D Abdulla A Zhang S Roller J Menger MD Regner S Lymphocyte function antigen-1 regulates neutrophil recruitment and tissue damage in acute pancreatitis. Br J Pharmacol (2011) 163:413–23. 10.1111/j.1476-5381.2011.01225.x 21244370 PMC3087141

[4] Awla D Hartman H Abdulla A Zhang S Rahman M Regner S Rho-kinase signalling regulates trypsinogen activation and tissue damage in severe acute pancreatitis. Br J Pharmacol (2011) 162:648–58. 10.1111/j.1476-5381.2010.01060.x 20942858 PMC3041254

[5] Xiang H Zhang Q Qi B Tao X Xia S Song H Chinese herbal medicines attenuate acute pancreatitis: pharmacological activities and mechanisms. Front Pharmacol (2017) 8:216. 10.3389/fphar.2017.00216 28487653 PMC5403892

[6] Maleth J Rakonczay Z Jr Venglovecz V Dolman NJ Hegyi P. Central role of mitochondrial injury in the pathogenesis of acute pancreatitis. Acta Physiol (2013) 207:226–35. 10.1111/apha.12037 23167280

[7] Du D Jin T Zhang R Hu L Xing Z Shi N Phenolic compounds isolated from dioscorea zingiberensis protect against pancreatic acinar cells necrosis induced by sodium taurocholate. Bioorg Med Chem Lett (2017) 27:1467–70. 10.1016/j.bmcl.2017.01.014 28228364

[8] Malet H Canellas F Sawa J Yan J Thalassinos K Ehrmann M Newly folded substrates inside the molecular cage of the HtrA chaperone DegQ. Nat Struct Mol Biol (2012) 19:152–7. 10.1038/nsmb.2210 22245966 PMC3272482

[9] Hara K Shiga A Fukutake T Nozaki H Miyashita A Yokoseki A Association of HtrA1 mutations and familial ischemic cerebral small-vessel disease. N Engl J Med (2009) 360:1729–39. 10.1056/NEJMoa0801560 19387015

[10] Friedrich U Datta S Schubert T Plossl K Schneider M Grassmann F Synonymous variants in HtrA1 implicated in AMD susceptibility impair its capacity to regulate TGF-beta signalling. Hum Mol Genet (2015) 24:6361–73. 10.1093/hmg/ddv346 26310622

[11] Altobelli E Marzioni D Lattanzi A Angeletti PM HtrA1: its future potential as a novel biomarker for cancer. Oncol Rep (2015) 34:555–66. 10.3892/or.2015.4016 26035313 PMC4487665

[12] Teoh SS Zhao M Wang Y Chen Q Nie G. Serum HtrA1 is differentially regulated between early-onset and late-onset preeclampsia. Placenta (2015) 36:990–5. 10.1016/j.placenta.2015.07.001 26187609

[13] Rani R Smulian AG Greaves DR Hogan SP Herbert DR TGF-beta limits IL-33 production and promotes the resolution of colitis through regulation of macrophage function. Eur J Immunol (2011) 41:2000–9. 10.1002/eji.201041135 21469118 PMC3139176

[14] Shiga A Nozaki H Yokoseki A Nihonmatsu M Kawata H Kato T Cerebral small-vessel disease protein HtrA1 controls the amount of TGF-beta1 via cleavage of proTGF-beta1. Hum Mol Genet (2011) 20:1800–10. 10.1093/hmg/ddr063 21320870

[15] Iorio MV Croce CM. MicroRNAs in cancer: small molecules with a huge impact. J Clin Oncol (2009) 27:5848–56. 10.1200/JCO.2009.24.0317 19884536 PMC2793003

[16] Kusnierz-Cabala B Nowak E Sporek M Kowalik A Kuzniewski M Enguita FJ Serum levels of unique miR-551-5p and endothelial-specific miR-126a-5p allow discrimination of patients in the early phase of acute pancreatitis. Pancreatology (2015) 15:344–51. 10.1016/j.pan.2015.05.475 26094040

[17] Dixit AK Sarver AE Yuan Z George J Barlass U Cheema H Comprehensive analysis of microRNA signature of mouse pancreatic acini: overexpression of miR-21-3p in acute pancreatitis. Am J Physiol Gastrointest Liver Physiol (2016) 311:G974–80. 10.1152/ajpgi.00191.2016 27686613 PMC5130546

[18] Zhang XX Deng LH Chen WW Shi N Jin T Lin ZQ Circulating microRNA 216 as a marker for the early identification of severe acute pancreatitis. Am J Med Sci (2017) 353:178–86. 10.1016/j.amjms.2016.12.007 28183420

[19] Zhao Y Wang H Lu M Qiao X Sun B Zhang W Pancreatic acinar cells employ miRNAs as mediators of intercellular communication to participate in the regulation of pancreatitis-associated macrophage activation. Mediators Inflamm (2016) 2016:6340457. 10.1155/2016/6340457 27546996 PMC4980583

[20] Bazzoni F Rossato M Fabbri M Gaudiosi D Mirolo M Mori L Induction and regulatory function of miR-9 in human monocytes and neutrophils exposed to proinflammatory signals. Proc Natl Acad Sci U S A (2009) 106:5282–7. 10.1073/pnas.0810909106 19289835 PMC2664036

[21] Chen Z Chen Y Pan L Li H Tu J Liu C Dachengqi decoction attenuates inflammatory response via inhibiting HMGB1 mediated NF-kappaB and p38 MAPK signalling pathways in severe acute pancreatitis. Cell Physiol Biochem (2015) 37:1379–89. 10.1159/000430403 26488574

[22] Yao WY Zhou YF Qian AH Zhang YP Qiao MM Zhai ZK Emodin has a protective effect in cases of severe acute pancreatitis via inhibition of nuclear factorkappab activation resulting in antioxidation. Mol Med Rep (2015) 11:1416–20. 10.3892/mmr.2014.2789 25351888

[23] Zhang JW Zhang GX Chen HL Liu GL Owusu L Wang YX Therapeutic effect of Qingyi decoction in severe acute pancreatitis-induced intestinal barrier injury. World J Gastroenterol (2015) 21:3537–46. 10.3748/wjg.v21.i12.3537 25834318 PMC4375575

[24] Guo J Li W Shi H Xie X Li L Tang H Synergistic effects of curcumin with emodin against the proliferation and invasion of breast cancer cells through upregulation of miR-34a. Mol Cell Biochem (2013) 382:103–11. 10.1007/s11010-013-1723-6 23771315

[25] Lin SZ Xu JB Ji X Chen H Xu HT Hu P Emodin inhibits angiogenesis in pancreatic cancer by regulating the transforming growth factor-beta/drosophila mothers against decapentaplegic pathway and angiogenesis-associated microRNAs. Mol Med Rep (2015) 12:5865–71. 10.3892/mmr.2015.4158 26238071

[26] Si L Xu L Yin L Qi Y Han X Xu Y Potent effects of dioscin against pancreatic cancer via miR-149-3p-mediated inhibition of the Akt1 signalling pathway. Br J Pharmacol (2017) 174:553–68. 10.1111/bph.13718 28095588 PMC5345629

[27] Xiang H Zhang QK Wang DQ Xia SL Wang GJ Wu YJ iTRAQ-based quantitative proteomic analysis for identification of biomarkers associated with emodin against severe acute pancreatitis in rats. RSC Adv (2016) 6:72447–57. 10.1039/c6ra16446c

[28] Rizvi IA Robinson K McFadden DW Riggs DR Jackson BJ Vona-Davis L. Peptide YY reverses TNF-alpha induced transcription factor binding of interferon regulatory factor-1 and p53 in pancreatic acinar cells. J Surg Res (2006) 136:25–30. 10.1016/j.jss.2006.05.028 16978650

[29] Xiang H Wang G Qu J Xia S Tao X Qi B Yin-Chen-Hao Tang attenuates severe acute pancreatitis in rat: an experimental verification of in silico network target prediction. Front Pharmacol (2016) 7:378. 10.3389/fphar.2016.00378 27790147 PMC5061810

[30] Choi SB Bae GS Jo IJ Wang S Song HJ Park SJ Berberine inhibits inflammatory mediators and attenuates acute pancreatitis through deactivation of JNK signalling pathways. Mol Immunol (2016) 74:27–38. 10.1016/j.molimm.2016.04.011 27148818

[31] Tenner S Baillie J DeWitt J Vege SS American College of Gastroenterology. American college of gastroenterology guideline: management of acute pancreatitis. Am J Gastroenterol (2013) 108(1400–15):1416. 10.1038/ajg.2013.218 23896955

[32] Polito F Bitto A Irrera N Squadrito F Fazzari C Minutoli L Flavocoxid, a dual inhibitor of cyclooxygenase-2 and 5-lipoxygenase, reduces pancreatic damage in an experimental model of acute pancreatitis. Br J Pharmacol (2010) 161:1002–11. 10.1111/j.1476-5381.2010.00933.x 20977452 PMC2998682

[33] Jakkampudi A Jangala R Reddy BR Mitnala S Nageshwar Reddy D Talukdar R NF-kappaB in acute pancreatitis: mechanisms and therapeutic potential. Pancreatology (2016) 16:477–88. 10.1016/j.pan.2016.05.001 27282980

[34] Wang G Liu Y Zhou SF Qiu P Xu L Wen P Effect of somatostatin, ulinastatin and gabexate on the treatment of severe acute pancreatitis.Am J Med Sci (2016) 351:506–12. 10.1016/j.amjms.2016.03.013 27140710

[35] He Y Wu CF Li JH Li HL Sun ZH Zhang H Inulin-type fructans modulates pancreatic-Gut innate immune responses and Gut Barrier integrity during experimental acute pancreatitis in a chain length-dependent manner. Front Immunol (2017) 8:1209. 10.3389/fimmu.2017.01209 29018453 PMC5622924

[36] Schmitz J Owyang A Oldham E Song Y Murphy E McClanahan TK IL-33, an interleukin-1-like cytokine that signals via the IL-1 receptor-related protein ST2 and induces t helper type 2-associated cytokines. Immunity (2005) 23:479–90. 10.1016/j.immuni.2005.09.015 16286016

[37] Saluja R Khan M Church MK Maurer M. The role of IL-33 and mast cells in allergy and inflammation. Clin Transl Allergy (2015) 5:33. 10.1186/s13601-015-0076-5 26425339 PMC4588911

[38] Ndaw VS Abebayehu D TGF-beta1 suppresses IL-33-induced mast cell function. J Immunol (2017) 199:866–73. 10.4049/jimmunol.1601983 28637902 PMC5538185

