# Supplementary material for: Emodin Alleviates Sodium Taurocholate-Induced Pancreatic Acinar Cell Injury via MicroRNA-30a-5p-Mediated Inhibition of High-Temperature Requirement A/Transforming Growth Factor Beta 1 Inflammatory Signaling
Source: Front Immunol. 2017 Nov 6;8:1488. doi: 10.3389/fimmu.2017.01488 (PMC5681496; doi:10.3389/fimmu.2017.01488)
Supplement: Supplementary file 1 [file data_sheet_1.docx]

**Emodin alleviates sodium taurocholate-induced pancreatic acinar cell injury via microRNA-30a-5p mediated inhibition of HTRA/TGF-β1 inflammatory signalling**

**Supporting Information**

***Hong Xiang^1, 2^, Xufeng Tao^3^, Shilin Xia^4^, Jialin Qu^4^, Huiyi Song^4^, Jianjun Liu^4^, Dong Shang^1, 2*^***

1. *College (Institute) of Integrative Medicine, Dalian Medical University, Dalian 116044, China.*
2. *Department of General Surgery, the First Affiliated Hospital of Dalian Medical University, Dalian 116011, China.*
3. *College of Pharmacy, Dalian Medical University, Dalian 116044, China.*
4. *Clinical Laboratory of Integrative Medicine, the First Affiliated Hospital of Dalian Medical University, Dalian 116011, China.*

**To Corresponding author,*

*Dr. Dong Shang*

*Department of General Surgery, Pancreatico-Biliary Center*

*The First Affiliated Hospital of Dalian Medical University*

*Dalian, China*

*College (Institute) of Integrative Medicine*

*Dalian Medical University*

*Dalian, China.*

*Tel: +86-411-83635963*

*Fax: +86-411-83622844*

*E-mail: shangdong@dmu.edu.cn*

**Table S1.** Prospective target miRNAs of HTRA1 via bioinformatics approaches

| miRNA | ID | miRanda | miRDB | TargetScan | CLIP |
| --- | --- | --- | --- | --- | --- |
| rno-miR-323-3p | MIMAT0000550 | 1 | 1 | 1 | 0 |
| rno-miR-329-3p | MIMAT0000566 | 1 | 0 | 1 | 0 |
| rno-miR-665 | MIMAT0012844 | 1 | 0 | 1 | 0 |
| rno-miR-3552 | MIMAT0017813 | 0 | 1 | 1 | 0 |
| rno-miR-494-3p | MIMAT0003193 | 1 | 0 | 1 | 0 |
| rno-miR-345-5p | MIMAT0000594 | 1 | 0 | 1 | 0 |
| rno-miR-30a-5p | MIMAT0000808 | 1 | 0 | 1 | 0 |
| rno-miR-628 | MIMAT0012836 | 1 | 0 | 1 | 0 |

**Table S2.** The information of the sequences used in the present work.

| Name | Sequences |
| --- | --- |
| miR-30a-5p primer | 5’-UGUAAACAUCCUCGACUGGAAG-3’ |
| miR-30a-5p mimic | 5’-UGUAAACAUCCUCGACUGGAAG-3’ |
|  | 3’-ACAUUUGUAGGAGCUGACCUUC-5’ |
| miR-30a-5p inhibitor | 5’-CUUCCAGUCGAGGAUGUUUACA-3’ |
| miR-30a-5p antagomir | 5’-CUUCCAGUCGAGGAUGUUUACA-3’ |

**Table S3.** Histological scoring for pancreatitis.

| Condition | Score | Description |
| --- | --- | --- |
| Edema | 0  1  2  3  4 | Absent  Diffuse expansion of interlobular septa  1 + diffuse expansion of interlobular septa  2 + diffuse expansion of interlobular septa  3 + diffuse expansion of intercellular septa |
| Inflammation (%) | 0  1  2  3  4 | Absent  Around ductal margin  In parenchyma (<50 of lobules)  In parenchyma (51-75 of lobules)  In parenchyma (>75 of lobules) |
| Vacuolization (%) | 0  1  2  3  4 | Absent  Periductal (<5)  Focal (5-20)  Diffuse (21-50)  Severe (>50) |

**Table S4.** The primer sequences used for real-time PCR assay

| Gene | GenBank accession | Primers (5'-3') |
| --- | --- | --- |
| IL-6 | NM_012589.2 | Forward: CTGATTGTATGAACAGCGATGATG  Reverse: GGTAGAAACGGAACTCCAGAAGAC |
| IL-1β | NM_012589.2 | Forward: TCAGGAAGGCAGTGTCACTCA  Reverse:CATCATCCCACGAGTCACAGA |
| TNF-α | NM_012675.3 | Forward: CAAGAGCCCTTGCCCTAAGG  Reverse: CGGACTCCGTGATGTCTAAGTACTT |
| HTRA1 | NM_031721.1 | Forward:GCGAGTGGGTCAGGATTCAT  Reverse:AATGTCGGCCTTTTCATCCA |
| GAPDH | NM_017008.3 | Forward: GGCACAGTCAAGGCTGAGAATG  Reverse: ATGGTGGTGAAGACGCCAGTA |


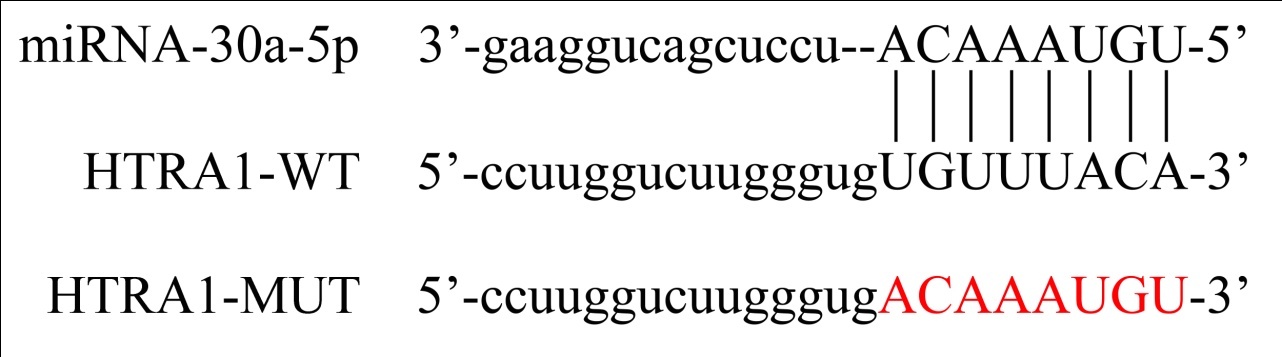


**Figure S1** RNA sequence alignment showed that the 3'-UTR of HTRA1 mRNA contained a site complementary to the seed region of miR-30a-5p.


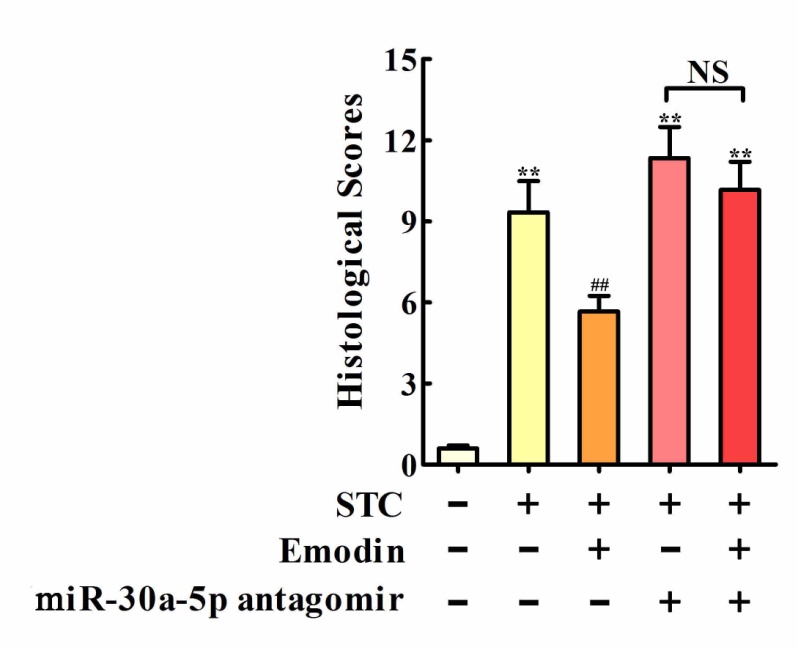


**Figure S2** Histological scoring for pancreatitis basing on the HE staining. Data are presented as the mean ± SD (n = 6), where 'n' refers to independent values. ^**^p < 0.01 versus control group; ^##^p < 0.01 versus model group; NS, not significant.
